# Longitudinal associations between neighborhood safety and adolescent adjustment: The moderating role of affective neural sensitivity

**DOI:** 10.1016/j.dcn.2024.101380

**Published:** 2024-04-12

**Authors:** Tianying Cai, Beiming Yang, Zexi Zhou, Ka I. Ip, Emma K. Adam, Claudia M. Haase, Yang Qu

**Affiliations:** a School of Education and Social Policy, Northwestern University, Evanston, IL, United States; b Department of Human Development and Family Sciences, University of Texas at Austin, Austin, TX, United States; c Institute of Child Development, University of Minnesota, Twin Cities, Minneapolis, MN, United States

**Keywords:** Neighborhood safety, Insula, Anterior cingulate cortex, Differential susceptibility, Mental health, Sleep

## Abstract

Research on social determinants of health has highlighted the influence of neighborhood characteristics (e.g., neighborhood safety) on adolescents’ health. However, it is less clear how *changes* in neighborhood environments play a role in adolescent development, and who are more sensitive to such changes. Utilizing the first three waves of data from the Adolescent Brain Cognitive Development (ABCD) project (*N* = 7932, *M* (*SD*) _age_ = 9.93 (.63) years at T1; 51% boys), the present study found that increases in neighborhood safety were associated with decreased adolescent externalizing symptoms, internalizing symptoms, but not sleep disturbance over time, controlling for baseline neighborhood safety. Further, adolescents’ insula and anterior cingulate cortex (ACC) reactivity to positive emotional stimuli moderated the association between changes in neighborhood safety and adolescent adjustment. Among youth who showed higher, but not lower, insula and ACC reactivity to positive emotion, increases in neighborhood safety were linked with better adjustment. The current study contributes to the differential susceptibility literature by identifying affective neural sensitivity as a marker of youth’s susceptibility to changes in neighborhood environment. The findings highlight the importance of neighborhood safety for youth during the transition to adolescence, particularly for those with heightened affective neural sensitivity.

## Highlights

• Increases in neighborhood safety contributed to better adjustment over time.
• Youth with higher affective neural reactivity are sensitive to neighborhood changes.
• Youth with lower affective neural sensitivity are less impacted by neighborhood.

Across the globe, there are enormous disparities in the kinds of neighborhoods youth grow up in (e.g., Lund et al., 2018). Within the United States, many forms of institutionalized injustice, discrimination, and racism have shaped neighborhood disparities (Lee et al., 2022), with some youth growing up in neighborhoods that are safe and protected and others experiencing unsafety and exposure to violence (Turner et al., 2013). Exposure to violence and lack of neighborhood safety have been found to predict worse adolescent adjustment (Baranyi et al., 2021, Mayne et al., 2021). At the same time, not all adolescents respond to their environments in the same way. This idea figures prominently in differential susceptibility models broadly and neurobiological susceptibility to social context models specifically (Ellis et al., 2011, Schriber and Guyer, 2016), which suggest adolescents with higher neurobiological sensitivity may be more susceptible to both positive and negative environments, leading to “for better and for worse” outcomes. Using data from a large-scale longitudinal study in the United States – the Adolescent Brain Cognitive Development (ABCD) study (Casey et al., 2018), we sought to investigate how baseline and changes in neighborhood safety were linked with adolescent adjustment during early adolescence. Moreover, building on neurobiological susceptibility to social context models (Ellis et al., 2011, Schriber and Guyer, 2016) and insights into the role of salience network in emotional responding (Seeley, 2019), we examined whether individual differences in neural reactivity to emotional stimuli in insula, anterior cingulate cortex (ACC), and amygdala would moderate the link between changes in neighborhood safety and adolescent adjustment.

Neighborhood characteristics are crucial for adolescent adjustment, with unique contributions beyond the more proximal family context (Heissel et al., 2018). Adolescents – who are moving through a period of critical neurophysiological and socioemotional changes – appear to be even more susceptible to neighborhood disadvantage than younger children (Alvarado, 2016). Given the importance of safety needs (Maslow, 1954), perceived neighborhood safety, which refers to families’ experience of security and vulnerability to crime and violence in the neighborhood (Hill et al., 2016), stands out as an important dimension. Lack of neighborhood safety is a source of stress for youth either through directly being the target of violence or through indirectly hearing and observing violence (Sharkey, 2018). Indeed, prior studies have linked the lack of neighborhood safety with more externalizing symptoms, internalizing symptoms, and sleep problems (Fowler et al., 2009, Mayne et al., 2021). While extant studies have provided important insights, they also often relied on cross-sectional designs and single-snapshots of neighborhood characteristics. Despite notable experimental studies identifying the health benefits of moving from a worse to a better neighborhood (Ludwig et al., 2011, Sanbonmatsu et al., 2012), longitudinal studies are needed to understand whether *changes* in neighborhood safety are linked with adjustments during the critical period of early adolescence (Mayne et al., 2021), especially in non-experimental settings where neighborhood environment changes happen in a more natural manner.

Moreover, some adolescents are more sensitive to their environments than others. The differential susceptibility to environment model proposes that youth with high neurobiological reactivity are sensitive to both positive and negative environments, while less sensitive youth are less affected by their environments (Belsky and Pluess, 2009, Ellis et al., 2011, Schriber and Guyer, 2016). Yet, a gap in the differential susceptibility literature remains about how youth with high neurobiological reactivity would be affected by the positive (e.g., increases in neighborhood safety) and negative (e.g., increases in neighborhood threat) *changes* in their surrounding environment. Given that adolescents’ neurobiological reactivity may influence how they perceive cues about safety and threat from the environment (Guyer, 2020), understanding how youth with different neurobiological reactivity perceive and respond to neighborhood changes can provide novel and important insights into the differential susceptibility theory.

Insula, ACC, and amygdala reactivity to emotional stimuli are potential markers of individual differential susceptibility to changes in the neighborhood environment. Emotions play a central role in mental health and facial expressions of emotion in particular serve important social signaling functions (Rodosky et al., 2023). Both the insula and ACC are major hubs of the salience network, which are involved in the processing of emotionally salient stimuli (Gasquoine, 2014, Menon, 2015, Seeley, 2019). As the primary region for reception of interoceptive inputs from the whole body, the insula integrates interoceptive stimuli with emotional states to form conscious experience of emotion (Craig, 2010, Damasio, 2003). Greater insula activation allows greater awareness of individuals’ emotional state and perception of bodily reactions to emotion-provoking objects (Acevedo et al., 2014, Craig, 2010). Furthermore, with connections to both the limbic system and prefrontal cortex, the ACC integrates neural circuitry for affect regulation, including emotion assessment, emotion-related regulation, and autonomic integration (Stevens et al., 2011). Prior studies suggested ACC and insula activation to social stimuli moderated the link between parenting and adolescent depression, such that youth with higher ACC and insula reactivity to social stimuli thrive under positive parenting but suffer under negative parenting (Rudolph et al., 2020). The amygdala has been identified as critical for emotion processing and salience detection, especially the processing of negative emotions (LeDoux, 2000). For example, recent research found that amygdala reactivity to emotional faces amplified the effects of family rearing experiences on youth’s internalizing symptoms, externalizing symptoms, and prosocial behaviors (Liu et al., 2021). In addition, research on adult samples also identified similar patterns, such that among individuals with higher amygdala reactivity to emotional faces, greater socioeconomic resources predicted less antisocial behavior two years later (Gard et al., 2018). Yet, it remains unclear whether insula, ACC, and amygdala reactivity to emotional stimuli may be associated with individual differential susceptibility to ecological context relevant to adolescents’ daily life (i.e., changes in neighborhood safety), especially during early adolescence.

## 1 The current study

**Fig. 1:**
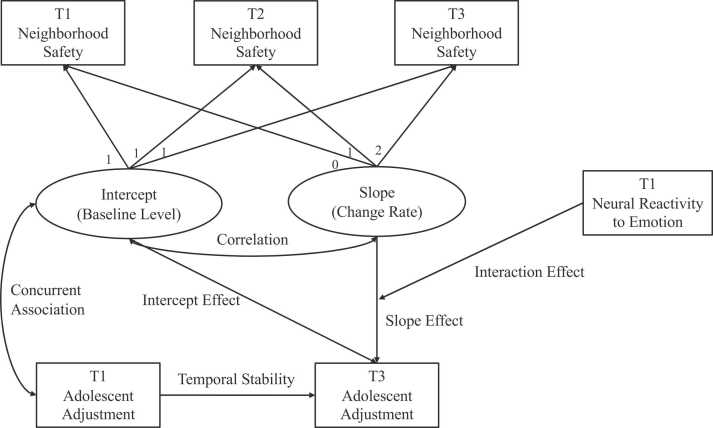
Conceptual Model. Note: In the actual analysis, all three adolescent adjustment outcomes (i.e., externalizing symptoms, internalizing symptoms, and sleep disturbance) are included in one model. For details, see Supplementary Table 1.

Using three-wave longitudinal data from the ABCD study, the current study has two aims. Our first aim was to examine whether baseline and changes in neighborhood safety predicted adolescent adjustment over time (Fig. 1), controlling for age, race, sex at birth, parental educational attainment, area deprivation index (ADI), family conflict, and baseline adjustment. Based on prior literature (Fowler et al., 2009), we hypothesized that both higher baseline and increases in neighborhood safety would predict decreases in internalizing symptoms, externalizing symptoms, and sleep disturbance two years later. Our second aim was to investigate whether insula, ACC, and amygdala reactivity to emotional stimuli (i.e., positive and negative emotions) moderated the association between changes in neighborhood safety and adolescent adjustment (Fig. 1). Based on prior evidence (Rudolph et al., 2020, Schriber and Guyer, 2016), we hypothesized that insula, ACC, and amygdala reactivity to emotional stimuli would moderate the association between changes in neighborhood safety and adolescent adjustment. Specifically, we expected that increases in neighborhood safety would be linked with better adolescent adjustment only among youth with higher insula, ACC, and amygdala reactivity. The study is not pre-registered.

## 2 Methods

### 2.1 Participants

Data were obtained from baseline (T1), one-year follow-up (T2), and two-year follow-up (T3) of the Adolescent Brain Cognitive Development (ABCD) study (data release 4.0). All the data included in the current study are available on the NIMH Data Archive (https://nda.nih.gov/abcd) upon data access request. Participants of the ABCD study were recruited at 21 sites in the United States using probability sampling (Garavan et al., 2018). Previous work documents a variety of measures that were used for this study, including task-based fMRI and behavioral outcomes (Casey et al., 2018). Among the full sample of 11,876 youth at T1, a total of 7932 youth (mean age = 9.93 years, *SD* = 0.63; 51% boys and 49% girls) and their primary caregivers (89% mothers and 11% fathers) were included in the analyses. The current research included participants based on the inclusion criteria provided by the ABCD team (i.e., participants with variable “imgincl_nback_include” = 1), which are the recommended quality control criteria of the Emotional n-back task in ABCD data release note 4.0 (e.g., passing the Emotional n-back task behavior, FreeSurfer quality control, and fMRI manual post-processing quality control; for detailed criteria, see ABCD Human Subjects Study, 2021). A comparison of participants with and without valid data for emotional n-back task revealed that, compared with participants without valid neuroimaging data, participants with valid data were older (*M*_included_ = 119.50 months, *M*_excluded_ = 117.93 months, *t* (8051.03) = −10.880, *p* <.001), had higher parental education (*M*_included_ = 3.89, *M*_excluded_ = 3.44, *t* (7148.23) = −19.448, *p* <.001), had lower family conflict (*M*_included_ = 1.94, *M*_excluded_ = 2.25, *t* (7497.37) = 7.91, *p* <.001), were more likely to be White and Asian (*χ*^2^ (4) = 375.39, *p* <.001), and lived in areas of lower area deprivation index (ADI; *M*_included_ = 37.35, *M*_excluded_ = 45.54, *t* (6338.89) = 14.38, *p* <.001).

### 2.2 Emotional N-Back Task

At T1, youth completed the Emotional n-back task during fMRI scanning. The Emotional n-back task is an fMRI paradigm including an active n-back working memory component and a passive emotional perception component (Casey et al., 2018). There were two runs of this task that contained eight blocks of trials and four 15-second rest periods each. Each block involved 10 trials that lasted 2.5 seconds. These 160 trials displayed 96 unique stimuli of 4 different stimulus types, which are negative-facial (fearful), positive-facial (happy), neutral-facial, and non-facial (places) stimuli. Each type of stimulus has the same number of trials. The task included two conditions, a 0-back and a 2-back condition. In each run, there are four blocks with the 0-back condition and four blocks with the 2-back condition. The 0-back condition requires a low memory load. In the 0-back condition, participants were instructed to respond whether a stimulus presented on the current screen matched the first screen they saw at the beginning of the block by indicating either “Match” or “No Match”. The 2-back condition requires a high memory load. In the 2-back condition, participants were instructed to respond if a facial stimulus on the screen matched the one shown two trials before by indicating either “Match” or “No Match”. The working memory component examined the contrast between the 0-back condition and the 2-back condition. The emotional perception component examined the contrast between emotional facial stimulus (i.e., positive or negative faces) and neutral facial stimulus. The current study focused on the emotional perception component of the Emotional n-back task, which was examined across conditions with both high memory load (i.e., the 2-back condition) and low memory load (i.e., the 0-back condition).

### 2.3 fMRI data acquisition and preprocessing

The ABCD study used a harmonized neuroimaging protocol across 21 sites. Three 3 T scanner platforms (i.e., Siemens Prisma [Siemens Healthineers], GE 750 [GE Healthcare], and Philips [Philips Healthcare]) were used. For Siemens scanners, the following scanning parameters were used for T1 structural image acquisition: matrix = 256×256, 176 slices, field of view (FOV) = 256×256, resolution (mm) = 1.0×1.0×1.0, repetition time (TR) = 2500 ms, echo time (TE) = 2.88 ms, inversion time (TI) = 1060 ms, flip angle = 8°. For Phillips scanners, the following scanning parameters were used for T1 structural image acquisition: matrix = 256×256, 225 slices, FOV = 256×240, resolution (mm) = 1.0×1.0×1.0, TR = 6.31 ms, TE = 2.9 ms, TI = 1060 ms, flip angle = 8°. For GE scanners, the following scanning parameters were used for T1 structural image acquisition: matrix = 256×256, 208 slices, FOV = 256×256, resolution (mm) = 1.0×1.0×1.0, TR = 2500 ms, TE = 2 ms, TI = 1060 ms, flip angle = 8°. Across all scanners, the following scanning parameters were used for T2* weighted functional images associated with the Emotional n-back task: matrix = 90×90, 60 slices, FOV = 216 × 216, TE/ TR (ms) = 800/30, flip angle = 52°, resolution (mm) = 2.4 × 2.4 × 2.4, multiband acceleration factor = 6. Each scanner used a standard head coil for the initial time point of fMRI data acquisition. The Emotional n-back task was presented to participants in a random order along with other functional tasks included in the study. E-Prime Professional software (Version 2.0; Schneider et al., 2012) was used to deliver the stimuli for the Emotional n-back task, and Current Designs button boxes were used to record responses (Science Plus Group, Groningen, The Netherlands).

Multimethod quality assurance checks looked for problems with acquisition, artifacts, motion, or file corruption (Casey et al., 2018). Subsequent preprocessing of these images removed initial frames of functional images, estimated within-volume head motion, and performed rigid body motion correction in each individual. Data were then processed for image distortions resulting from B0 field inhomogeneity. Isotropic resampling (2.4 mm) aligned fMRI data across participants from all sites before functional data was then registered to each individual’s T1w structural image. Following preprocessing, images were sampled onto the cortical surface of each subject using FreeSurfer functions (Hagler et al., 2019). General linear modeling using AFNI’s 3dDeconvolve (Cox, 1996) was used to calculate individual-level models. Baseline and quadratic trends in time-series data were included in the first-level analysis. Motion estimates and their derivatives were included as regressors of no interest (Power et al., 2014). In cases where a single time point was associated with FD greater than 0.9, this volume was censored. Estimates were filtered with an infinite impulse response notch filter, which attenuates signals in the range of 0.31–0.43 Hz. A two-parameter gamma basis function was convolved with onsets of the onset of all trials during the 0-back and 2-back conditions.

Given our interest in neural reactivity to emotional stimuli, the current study focused on the trials of positive and negative emotions in the Emotional n-back task. Reactivity to positive emotion was measured by the contrast between the trials of happy faces vs. the trials of neutral faces. Reactivity to negative emotion was measured by the contrast between the trials of fearful faces vs. the trials of neutral faces. Past research has highlighted insula, ACC, and amygdala (Schriber and Guyer, 2016, Seeley, 2019) as key neural regions involved in processing salient emotional stimuli. Therefore, the current study employed a region-of-interest approach by examining youth’s insula, ACC, and amygdala reactivity to positive and negative social stimuli. Using Freesurfer (Fischl, 2012), estimations of insula, ACC, and amygdala activity were derived by applying anatomically-defined parcellations (i.e., the Desikan-Killiany and Destrieux atlases) to each participant’s cortical surface space (Fischl et al., 2002).

### 2.4 Questionnaire measures

**Neighborhood Safety**. At each wave, neighborhood safety was assessed with the safety from the crime scale of the neighborhood characteristics measure (Echeverria, 2004). On a five-point Likert scale (1 = *strongly disagree* to 5 = *strongly agree*), parents responded to three items on whether their neighborhood was safe (i.e., “I feel safe walking in my neighborhood, day or night”, “Violence is not a problem in my neighborhood”, and “My neighborhood is safe from crime”). The mean was taken across all items, with a higher number indicating a safer neighborhood environment. This measure showed good internal consistency, with Cronbach’s Alphas (*α*s) = .88 at T1, .88 at T2, and .86 at T3.

**Youth’s Externalizing Symptoms.** At T1 and T3, youth’s externalizing symptoms were measured with the parent-reported Child Behavior Checklist (Achenbach, 1991). On a three-point Likert scale (0 = *not true* to 2 = *very true or very often*), parents reported on the youth’s externalizing symptoms (35 items on rule-breaking and aggressive behaviors, e.g., “breaks rules at home, school or elsewhere” and “demands a lot of attention”). For participants who have valid answers to all the items, the sum score of all items was converted to norm-referenced T-score, with a higher number indicating more externalizing symptoms among youth. This measure showed good internal consistency, with *α*s =.86 at T1 and .87 at T3.

**Youth’s Internalizing Symptoms.** At T1 and T3, youth’s internalizing symptoms were measured with the parent-reported Child Behavior Checklist (Achenbach, 1991). On a three-point Likert scale (0 = *not true* to 2 = *very true or very often*), parents reported on the youth’s internalizing symptoms (32 items on anxious depressive symptoms, withdrawn depressive symptoms, and somatic complaints, e.g., “unhappy, sad, or depressed” and “feels worthless or inferior”). For participants who have valid answers to all the items, the sum score of all items was converted to norm-referenced T-score, with a higher number indicating more internalizing symptoms among youth. This measure showed excellent internal consistency, with *α*s = .90 at T1 and .89 at T3.

**Youth’s Sleep Disturbance.** At T1 and T3, youth’s sleep disturbance was measured with the Sleep Disturbance Scale for Children (SDSC; Bruni et al., 1996). On a five-point Likert scale (1 = *never* to 5 = *always/daily*), parents reported on their youth’s sleep problems (26 items, e.g., “the child has difficulty getting to sleep at night” and “the child awakes in the morning feeling tired”). For participants who have valid answers to all the items, the sum score of all items was calculated, with a higher number indicating greater sleep disturbance among youth. This measure showed good internal consistency, with *α*s =.81 at T1 and .82 at T3.

**Demographic Covariates.** In line with prior research using the ABCD data, the current study included youth’s age, sex at birth, race, parents’ educational attainment, ADI, and youth-reported family conflict, as demographic covariates. Youth’s age was their age at the baseline assessment. Youth’s sex at birth was coded into 0 = *male* and 1 = *female*. Youth’s race was coded into five dummy variables each representing *White*, *Black*, *Latinx*, *Asian*, and *mixed/other races*. Parents’ educational attainment was the highest degree obtained by either the primary or secondary caregivers of the youth, ranging from 1 = *less than a high school diploma* to 5 = *postgraduate degree*. ADI represents neighborhood deprivation, which is calculated as a composite score based on 17 factors (e.g., income, education, housing) from the American Community Survey, with higher values indicating higher disadvantage (Kind and Buckingham, 2018). Youth-reported family conflict was measured by family conflict subscale of the family environment scale (Hoffman et al., 2019), with nine categorical items (e.g., we fight a lot in our family, 1 = *yes*, 0 = *no*). The sum of the items was calculated, with higher scores representing more family conflict.

### 2.5 Overview of the analyses

Pearson correlation and descriptive statistics were first examined before the primary analyses. Primary analyses were conducted in Mplus 8.6 (Muthén and Muthén, 2017). Maximum likelihood with robust standard errors (MLR) estimators were used, which is robust to nonnormality while handling missing data (Kline, 2005). Considering the nested nature of the ABCD dataset (i.e., participants recruited from 21 research sites and may include siblings from the same family), all structural equation models (SEM) were estimated using Taylor series linearization using Type = COMPLEX, with CLUSTER = rel_family_id to account for family units and STRATIFICATION = site_id_l to account for study sites. Model fit for SEM models was examined using the following criteria (Kline, 2005): comparative fit index (CFI) > .90, Tucker–Lewis index (TLI) > .90, root-mean-square error of approximation (RMSEA) < .08, standardized root mean squared residual (SRMR) < .08. All models were fitted including demographic variables as covariates (i.e., youth’s age, sex at birth, race, parental educational attainment, ADI, and family conflict).

The first aim was to examine how the baseline and changes in neighborhood safety predicted adolescent adjustment (i.e., externalizing symptoms, internalizing symptoms, and sleep disturbance) over two years in early adolescence, controlling for baseline adolescent adjustment and demographic covariates. Latent growth curve models (LGCM) were used to investigate interindividual differences in the developmental trajectories of neighborhood safety as well as the associations between these trajectories and adolescent adjustment (Preacher et al., 2008). First, an unconditional LGCM was fitted with the estimation of intercept (i.e., baseline level) and slope (i.e., growth rate or changes) of neighborhood safety over three time points. Upon adequate model fit, one conditional LGCM was fitted for baseline and changes in neighborhood safety predicting adolescent adjustment (i.e., externalizing symptoms, internalizing symptoms, and sleep disturbance), controlling for demographic variables and T1 adjustment (Fig. 1).

The second aim was to examine how neural reactivity to emotion may influence the association between changes in neighborhood safety and adolescent adjustment (Fig. 1). Latent interactions between the changes in neighborhood safety and neural reactivity to emotions were added to the model using the “XWITH” function under TYPE = RANDOM in Mplus. Chi-square-based model fit statistics are not available under TYPE = RANDOM. Models were fitted separately for different brain regions and lateralization (i.e., right insula, right ACC, right amygdala, left insula, left ACC, left amygdala). Models were also fitted separately for two valences of emotion presented in the emotion n-back task (i.e., fearful vs. neutral, happy vs. neutral). Significant interaction terms were probed using simple slopes analysis with moderators at +/- 1 SD from the mean (Aiken and West, 1991). Significant interactions were plotted using Matplotlib library in Python, based on the data and statistical coefficients obtained from Mplus. Multiple comparison was adjusted for six brain regions using the False Discovery Rate (FDR) method (Benjamini and Yekutieli, 2001) using the the “p.adjust (pvalues, method=‘BH’)” function in the ‘*stats*’ package in R so that only the interaction terms with p-values under .05 after FDR correction were considered significant.

To further understand whether the interaction patterns demonstrated differential susceptibility, and to distinguish interaction patterns that represent differential susceptibility from diathesis-stress (i.e., some more sensitive and some more resistant to the consequences of negative experiences and exposures) or vantage sensitivity (i.e., some more sensitive and some more resistant to the beneficial effects of positive experiences and exposures; Pluess, 2015), a series of post-hoc analysis was conducted following the guideline by Roisman et al. (2012) and Deane et al. (2020). First, region of significance on X (predictor) test was conducted, which determined the values of X (predictor) for which the regression of Y (outcome) on Z (moderator) was statistically significant. If the interaction indicates differential susceptibility, the association between Y and Z should be significant for both lower values of X (i.e., no more than 2 SD below the mean of X) and higher values of X (i.e., no more than 2 SD above the mean of X). Second, a region of significance on Z test was conducted, which determined the values of Z for which the regression of Y on X was statistically significant. If the interaction indicates differential susceptibility, the association between Y and X should be significant for values of Z that fall within 2 SDs of the mean of Z. Third, a Proportion of the Interaction (POI) index was calculated, which represented the proportion of the total interaction that were represented on the “better” side of the crossover point. A value close to 50% would indicate differential susceptibility while a value close to 0% or 100% would indicate diathesis-stress or vantage sensitivity, depending on the direction of the simple slope. Fourth, a Proportion Affected (PA) index was calculated, which represented the proportion of individuals (cases) for whom the effect of X on Y was “for better”. A non-trivial proportion would support differential susceptibility, while a value close to 0% or 100% would support diathesis-stress or vantage sensitivity, depending on the direction of the simple slope. For POI and PA, a cut-off of 20% was applied following the example by Deane et al. (2020), such that a POI/PA index between 20% - 80% would support differential susceptibility, while a POI/PA index between 0% - 20% and 80% - 100% would support diathesis-stress or vantage sensitivity (Deane et al., 2020, Roisman et al., 2012).

## 3 Results

Descriptive statistics and Pearson correlations of the primary study variables are reported in Table 1. Neighborhood safety at all timepoints was correlated with fewer externalizing symptoms, internalizing symptoms, and less sleep disturbance at both T1 and T3 (*r*s < -.07, *p*s <.001). Externalizing symptoms, internalizing symptoms, and sleep disturbance were positively correlated with each other at both T1 and T3 (*r*s >.41, *p*s <.001). Neighborhood safety was generally not correlated with insula,ACC, and amygdala reactivity to positive/negative emotion. Insula, ACC, and amygdala reactivity to positive emotion were generally not correlated with adolescent adjustment. Higher T1 insula, ACC, and amygdala reactivity to negative emotion were generally weakly correlated with fewer externalizing symptoms, internalizing symptoms, and sleep disturbance (Table 1).

**Table 1:** Descriptive statistics and correlations of primary study variables.

	1	2	3	4	5	6	7	8	9
1. T1 neighborhood safety	--								
2. T2 neighborhood safety	.63***	--							
3. T3 neighborhood safety	.59***	.62***	--						
4. T1 externalizing symptoms	-.12***	-.10***	-.11***	--					
5. T3 externalizing symptoms	-.07***	-.10***	-.12***	.69***	--				
6. T1 internalizing symptoms	-.11***	-.10***	-.09***	.59***	.43***	--			
7. T3 internalizing symptoms	-.08***	-.09***	-.10***	.43***	.57***	.64***	--		
8. T1 sleep disturbance	-.13***	-.10***	-.12***	.45***	.36***	.51***	.41***	--	
9. T3 sleep disturbance	-.09***	-.09***	-.11***	.36***	.41***	.39***	.47***	.61***	--
10. T1 right insula positive emotion	-.01	.01	.01	.00	-.02	-.01	-.01	-.01	.00
11. T1 right ACC positive emotion	-.01	.00	.00	.01	-.02	.00	-.03*	.01	-.01
12. T1 right amygdala positive emotion	-.01	.01	.00	-.01	-.03*	.00	-.01	.00	-.01
13. T1 left insula positive emotion	.00	.01	.01	.00	-.02	-.01	-.02	-.01	-.01
14. T1 left ACC positive emotion	-.01	-.01	-.01	.01	-.01	.00	-.03	.01	-.01
15. T1 left amygdala positive emotion	-.01	.01	.01	.00	-.02	.01	-.01	-.01	.00
16. T1 right insula negative emotion	.01	.02	.02	-.02	-.03*	-.02	-.02	-.03**	-.03*
17. T1 right ACC negative emotion	.00	.01	.02	-.01	-.05***	-.02	-.05***	-.02	-.03**
18. T1 right amygdala negative emotion	.00	.02	.01	-.03**	-.04**	-.02	-.01	-.02	-.02
19. T1 left insula negative emotion	.01	.02*	.02	-.02	-.03*	-.01	-.02	-.02	-.03*
20. T1 left ACC negative emotion	.00	.01	.01	-.01	-.03*	-.02	-.03*	-.02*	-.03*
21. T1 left amygdala negative emotion	.01	.01	.02	-.01	-.02	-.01	.00	-.02	-.02
Mean	3.95	3.94	3.92	45.03	43.98	48.17	47.76	36.21	36.19
SD	.93	.91	.91	10.02	9.48	10.43	10.35	7.73	7.76

	10	11	12	13	14	15	16	17	18	19	20	21

10. T1 right insula positive emotion	--											
11. T1 right ACC positive emotion	.66***	--										
12. T1 right amygdala positive emotion	.53***	.45***	--									
13. T1 left insula positive emotion	.82***	.67***	.47***	--								
14. T1 left ACC positive emotion	.66***	.89***	.41***	.69***	--							
15. T1 left amygdala positive emotion	.46***	.37***	.56***	.50***	.39***	--						
16. T1 right insula negative emotion	.43***	.25***	.18***	.38***	.27***	.19***	--					
17. T1 right ACC negative emotion	.28***	.41***	.12***	.31***	.37***	.14***	.64***	--				
18. T1 right amygdala negative emotion	.18***	.10***	.42***	.18***	.10***	.24***	.47***	.30***	--			
19. T1 left insula negative emotion	.32***	.21***	.15***	.43***	.24***	.19***	.82***	.66***	.42***	--		
20. T1 left ACC negative emotion	.26***	.32***	.09***	.31***	.40***	.13***	.65***	.87***	.32***	.69***	--	
21. T1 left amygdala negative emotion	.20***	.13***	.28***	.22***	.13***	.46***	.41***	.30***	.54***	.46***	.33***	

Mean	-.01	-.01	.00	-.02	-.01	-.01	.01	-.02	.07	.01	-.02	.05
SD	.35	.45	.50	.33	.42	.46	.33	.42	.46	.33	.41	.47

*Note*. ACC = anterior cingulate cortex.

* *p* <.05.

** *p* <.01.

*** *p* <.001.

### 3.1 Longitudinal link between neighborhood safety and adolescent adjustment

The first set of analyses examined whether baseline levels and changes in neighborhood safety predict adolescent adjustment (i.e., externalizing symptoms, internalizing symptoms, and sleep disturbance) over time. First, an unconditional latent growth curve model was fitted for neighborhood safety, which estimated its intercept and slope over the 3 years. Fit indices suggested good fit (CFI > .99; TLI > .99; RMSEA < .001, SRMR = .002). On average, parents reported a baseline neighborhood safety of 3.95 on a scale from 1 to 5, and a slight decrease in neighborhood safety over time (*M* (*SD*) = -.02 (.01), *p* < .001). The variances of intercept and slope were significant (σ^2^_Intercept_ = .58, *p* < .001; σ^2^_Slope_ = .03, *p* =.006), suggesting adequate interindividual differences in the baseline level and changes across time. The correlation between intercept and slope was significant (*B* = −.04, *SE* =.01, β = -.29, *p* = .002), suggesting that higher baseline levels of neighborhood safety were associated with less increases in neighborhood safety.

Next, one conditional latent growth curve model was fitted to examine the effects of baseline levels and changes in neighborhood safety on externalizing symptoms, internalizing symptoms, and sleep disturbance over two years, controlling for youth’s age, sex at birth, race, parents’ educational attainment, ADI, family conflict, and corresponding baseline adolescent adjustment (Table 2; Supplementary Figure 1). Model showed adequate model fit (CFI = .964, TLI = .914, RMSEA = .041, SRMR = .049). Increases in neighborhood safety were significantly associated with decreased externalizing symptoms (*B* = -9.44, *SE* = 3.86, β = -.16, *p* = .015), internalizing symptoms (*B* = - 5.84, *SE* = 2.87, β = -.09, *p* = .042), and not associated with decreased sleep disturbance (*B* = -3.14, *SE* = 1.95, β = -.06, *p* = .107) while controlling for baseline neighborhood safety. Further, higher baseline levels of neighborhood safety were associated with decreases in externalizing symptoms (*B* = -.65, *SE* = .21, β = -.05, *p* = .002), internalizing symptoms (*B* = -.97, *SE* = .21, β = -.07, *p* < .001), and sleep disturbance (*B* = -.56, *SE* = .16, β = -.06, *p* < .001) over time.

**Table 2:** Latent Growth Curve Model of neighborhood safety predicting adolescent adjustment.

	**Externalizing Symptoms**			**Internalizing Symptoms**			**Sleep Disturbance**		
	*B*	*SE*	β	*B*	*SE*	β	*B*	*SE*	β
**Predictor**									
Intercept (Initial Level of Neighborhood Safety)	-.65**	.21	-.05	-.97***	.21	-.07	-.56***	.16	-.06
Slope (Changes of Neighborhood Safety)	-9.44*	3.86	-.16	-5.84*	2.87	-.09	-3.14	1.95	-.06
***Covariates***									
T1 Adolescent Adjustment	.61***	.01	.66	.59***	.01	.61	.59***	.02	.60
Race-Black	-.47	.48	-.02	-2.22***	.48	-.07	-.98**	.36	-.04
Race-Latino	.10	.31	.00	-.45	.33	-.02	-.30	.22	-.02
Race-Asian	-.68	.68	-.01	-1.23	.78	-.02	-.38	.50	-.01
Race-Other	.14	.37	.01	.90*	.41	.03	.32	.28	.01
Age	.02	.01	.02	-.01	.01	-.01	.04***	.01	.04
Sex at birth	-.44*	.21	-.02	.38	.22	.02	.04	.15	.00
Parental Educational Attainment	.28*	.12	.03	.40**	.13	.04	.21*	.09	.03
ADI	.01*	.01	.04	.01	.01	.03	.01	.00	.02
Family Conflict	.10	.06	.02	.00	.06	.00	.04	.04	.01
***Model Fit***									
RMSEA	.041								
CFI	.964								
TLI	.914								
SRMR	.049								

*Note*: All three adjustment outcomes are included in one model.

* *p* <.05.

** *p* <.01.

*** *p* <.001.

### 3.2 Neural reactivity to emotion moderates the link between changes in neighborhood safety and adolescent adjustment

**Fig. 2:**
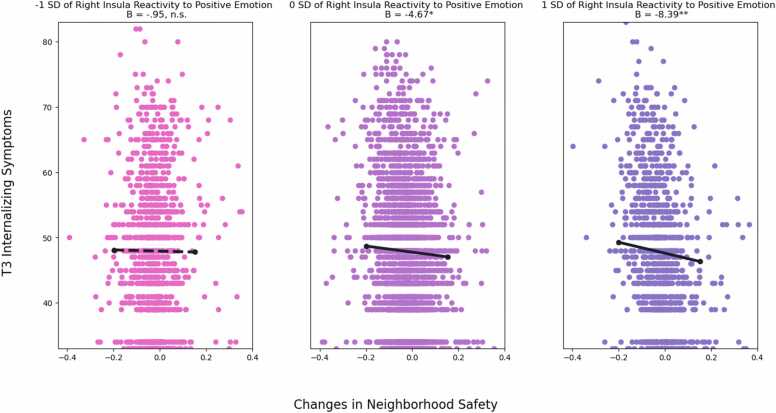
Right Insula Reactivity to Positive Emotion Moderates the Link between Changes in Neighborhood Safety and Internalizing Symptoms *Note*: Unstandardized coefficients are presented. Solid line represents significant slope, dashed line represents non-significant slope. Model controlled for baseline neighborhood safety, T1 internalizing symptoms, age, sex at borth, race, parental educational attainment, area deprivation index, and family conflict. * *p* <.05. ^**^p <.01.

The second set of analyses examined the moderating role of neural reactivity to emotion in the longitudinal association between changes in neighborhood safety and adolescent adjustment. Right insula reactivity to positive emotion moderated the interaction between trajectories of neighborhood safety and internalizing symptoms (*B* = -10.71, *SE* = 4.42, β = -.06, *p* = .015) and sleep disturbance (*B* = -7.39, *SE* = 2.68, β = -.06, *p* = .006), but not externalizing symptoms (*B* = -6.61, *SE* = 3.73, β = -.04, *p* = .076). The interactions in predicting internalizing symptoms and sleep disturbance remained significant after FDR correction for multiple comparisons of six brain regions (internalizing symptoms: FDR adjusted *p* = .045; sleep disturbance: FDR adjusted *p* = .036). As shown in Fig. 2, simple slope analyses revealed that among youth who showed higher levels of right insula reactivity to positive emotion (i.e., 1 SD above the mean), increases in neighborhood safety were associated with decreases in internalizing symptoms (*B* = -8.39, *SE* = 2.52, *p* = .001). However, among youth who showed lower levels of right insula reactivity to positive emotion (i.e., 1 SD below the mean), the association between changes in neighborhood safety and internalizing symptoms was not significant (*B* = -.95, *SE* = 2.23, *p* = .670). Region of significance on X test revealed that the association between right insula reactivity and internalizing symptoms was significant when changes in neighborhood safety were 1.06 SD below or .30 SD above the mean. Region of significance on Z test revealed that the association between changes in neighborhood safety and internalizing symptoms was significant when right insula reactivity was above -.29 SD of the mean. This interaction has a POI index of 55% and a PA index of 62%. Thus, this pattern of interaction demonstrated differential susceptibility (Roisman et al., 2012).

**Fig. 3:**
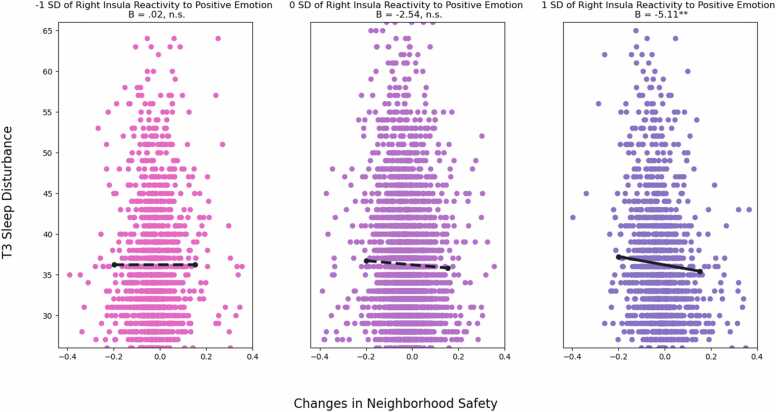
Right Insula Reactivity to Positive Emotion Moderates the Link between Changes in Neighborhood Safety and Predicting Sleep Disturbance *Note*: Unstandardized coefficients are presented. Solid line represents significant slope, dashed line represents non-significant slope. Model controlled for baseline neighborhood safety, T1 sleep disturbance, age, sex at birth, race, parental educational attainment, area deprivation index, and family conflict. ^**^p <.01.

Similarly, as shown in Fig. 3, increases in neighborhood safety were associated with decreased sleep disturbance over two years (*B* = -5.11, *SE* = 1.75, *p* = .003) among youth who showed higher levels of right insula reactivity to positive emotion, but this association was not significant among youth who showed lower levels of right insula reactivity to positive emotion (*B* = .02, *SE* = 1.58, *p* = .988). Region of significance on X test revealed that the association between right insula reactivity and sleep disturbance was significant when changes in neighborhood safety were .32 SD below or .64 SD above the mean. Region of significance on Z test revealed that the association between changes in neighborhood safety and sleep disturbance was significant when right insula reactivity was above .09 SD of the mean. This interaction had a POI index of 45% and a PA index of 36%. Thus, this pattern of interaction demonstrates differential susceptibility (Roisman et al., 2012).

**Fig. 4:**
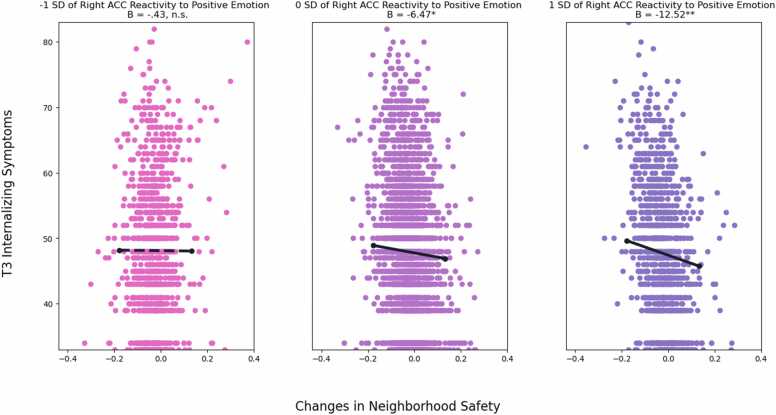
Right ACC Reactivity to Positive Emotion Moderates the Link between Changes in Neighborhood Safety and Internalizing Symptoms *Note*: Unstandardized coefficients are presented. Solid line represents significant slope, dashed line represents non-significant slope. Model controlled for baseline neighborhood safety, T1 internalizing symptoms, age, sex at birth, race, parental educational attainment, area deprivation index, and family conflict. * *p* <.05. ^**^p <.01.

Next, we examined the moderating role of ACC reactivity in the longitudinal link between changes in neighborhood safety and adolescent adjustment. Right ACC reactivity to positive emotion moderated the association between trajectories of neighborhood safety and internalizing symptoms (*B* = -13.47, *SE* = 5.42, β = -.09, *p* = .013) but not externalizing symptoms (*B* = -4.83, *SE* = 3.55, β = -.04, *p* = .174) or sleep disturbance (*B* = -2.95, *SE* = 2.69, β = -.03, *p* = .272). The interaction in predicting internalizing symptoms remained significant after FDR correction for multiple comparisons of six brain regions (FDR adjusted *p* = .045). Simple slope analyses revealed that (Fig. 4), among youth who showed higher levels of right ACC reactivity to positive emotion (i.e., 1 SD above the mean), increases in neighborhood safety were associated with decreases in internalizing symptoms (*B* = -12.52, *SE* = 4.30, *p* = .004). Yet, among youth who showed lower levels of right ACC reactivity to positive emotion (i.e., 1 SD below the mean), the association between changes in neighborhood safety and internalizing symptoms was not significant (*B* = -.43, *SE* = 2.88, *p* = .882). Supplementary Tables 1 and 2 Region of significance on X test revealed that the association between right ACC reactivity and internalizing symptoms was significant when changes in neighborhood safety were 1.40 SD below or .08 SD above the mean. Region of significance on Z test revealed that the association between changes in neighborhood safety and internalizing symptoms was significant when right ACC reactivity was above -.11 SD of the mean. This interaction had a POI index of 71% and a PA index of 75%. Thus, this pattern of interaction demonstrated differential susceptibility (Roisman et al., 2012). Further, no moderation effect was found for left insula or ACC reactivity to positive emotion, as well as insula and ACC reactivity to negative emotion (for details, see Supplementary Table 1 and 2). We also did not find any evidence that amygdala reactivity to emotion moderated the association between changes in neighborhood safety and adolescent adjustment (for details, see Supplementary Table 1 and Supplementary Table 2).

### 3.3 Sensitivity analyses

Three sets of sensitivity analyses were conducted. First, with prior literature highlighting the importance of right anterior insula in emotion regulation (Craig, 2009, Craig, 2010), this study explored if the moderation effect was evident for different sub-regions of insula based on the Destrieux atlases. Results revealed that the moderation effect was evident for right anterior insula (*B* = -8.48, *SE* = 3.90, β = -.06, *p* = .030), right inferior insula (*B* = -11.39, *SE* = 5.42, β = -.07, *p* = .036), right long insular gurus and central sulcus (*B* = -8.61, *SE* = 4.19, β = -.06, *p* = .040), and right short insular gyri (*B* = -6.59, *SE* = 2.98, β = -.05, *p* = .027) predicting internalizing symptoms, and for right anterior insula (*B* = -3.82, *SE* = 1.92, β = -.04, *p* = .047), right long insular gyrus and central sulcus of the insula (*B* = -6.94, *SE* = 3.12, β = -.06, *p* = .026), and right short insular gyri (*B* = -4.70, *SE* = 2.29, β = -.04, *p* = .040) predicting sleep disturbance (Supplementary Table 3). Second, there were a number of cases with neural reactivity beyond +/- 3 SD. Although such cases are generally normal and valuable given the large sample size (Saragosa-Harris et al., 2022), it is important to understand if those values have a vital influence on model outcomes. Thus, models were fit with insula, ACC, and amygdala reactivity scores beyond +/- 3 SD winsorized to +/- 3 SD. No meaningful changes in results were found (Supplementary Table 4). Third, the ABCD study has youth-reported internalizing and externalizing symptoms measured by the Brief Problem Monitor (BPM) in their two-year follow-up assessment (i.e., T3 in the current study) but not at the baseline assessment (i.e., T1 in the current study). Although the youth-reported outcomes and the analyses with these outcomes were *fundamentally different* (for details, see Supplementary Tables page 5) from our main analyses that used CBCL, a set of sensitivity analyses was conducted to explore whether the main or interaction effects were evident for youth reports. No main or interaction effects were found (for details, see Supplementary Table 5–7).

## 4 Discussion

Context matters in human development (Bronfenbrenner, 1979). A mounting body of empirical work has provided important insights into how micro-contexts (e.g., family and peer relationships) shape child and adolescent development. Research has also started to document the important role that more distal contexts (e.g., neighborhoods) play in shaping adolescents’ development (Fowler et al., 2009, Mayne et al., 2021). Yet it remains unclear how the longitudinal changes in neighborhood characteristics influence adolescent development (Chiang and Bai, 2021; Mayne et al., 2021), as well as how individual differences in neural responses play a role in this process. Using a large-scale, longitudinal sample of early adolescents, the study found that longitudinal increases in neighborhood safety were associated with decreases in externalizing symptoms and internalizing symptoms, but not sleep disturbance, while controlling for baseline neighborhood safety. We also identified adolescents’ right insula and ACC reactivity to positive emotion as a potential marker of differential susceptibility. Among youth with higher levels of right insula and ACC reactivity to positive emotion, increases in neighborhood safety were linked with fewer internalizing symptoms and less sleep disturbance, but decreases in neighborhood safety were linked with more internalizing symptoms and sleep disturbance. Conversely, among youth exhibiting lower right insula and ACC reactivity to positive emotion, the associations between changes in neighborhood safety and adolescent adjustment were not significant.

In line with our hypothesis, when examining *changes* in neighborhood safety, we found that both internalizing and externalizing symptoms (albeit not sleep disturbances) decreased when perceived safety increased over the two years, controlling for baseline neighborhood safety. This link between improvements in neighborhood safety and improvements in adolescents’ mental health highlights the malleability of adolescents’ mental health. These findings also underscore the potential downstream benefits of initiatives that seek to redress the impact of historical injustice in the U.S. (Egede et al., 2023). Findings are consistent with prior literature which documented the consistent link between neighborhood disadvantage and externalizing symptoms, as well as a smaller but significant link between neighborhood disadvantage and internalizing symptoms (Fowler et al., 2009). Living in a neighborhood that is perceived as unsafe can serve as a stressor in adolescents’ everyday life, leading to increases in psychological distress and triggering a state of arousal, which can result in elevated externalizing and internalizing symptoms and sleep disturbance (Mayne et al., 2021).

Moving beyond the link between neighborhood safety and adolescent adjustment, we found that the link between changes in neighborhood safety and internalizing symptoms was moderated by adolescents’ right insula and ACC reactivity to positive emotional stimuli. As core parts of the salience network, insula and ACC are often found to coactivate in response to emotionally salient stimuli (Gasquoine, 2014). The findings are in line with the differential susceptibility model (Belsky and Pluess, 2009; Ellis et al., 2011), such that heightened insula and ACC reactivity to positive emotion can be “for better and for worse” when adolescents are faced with changes in neighborhood safety. Similarly, the findings support the conceptual framework that individual differences in brain development can be susceptibility factors regarding the influence of social contexts on adolescent adjustment (Guyer, 2020, Schriber and Guyer, 2016). Given insula and ACC’s critical roles in the conscious experience of emotion (Craig, 2010, Damasio, 2003), adolescents with heightened affective reactivity in these regions may be more attuned to the emotion-relevant cues in the neighborhood environment. Thus, when the neighborhood they live in becomes safer, they may be more likely to pick up the positive cues about the improvement in the neighborhood environment, leading to better mental health and sleep quality. On the other hand, when the neighborhood becomes less safe, they may be also more likely to notice the lack of positive cues, leading to worse adjustment. Our study fills an important gap in the differential susceptibility research such that most research took a static view of environmental influences – that is, to understand how environments at baseline influence youth adjustment concurrently or longitudinally. However, the environment can change over time. Thus, in line with the differential susceptibility theory, youth with heightened affective neural sensitivity would be affected by both the increases in neighborhood safety (positive influences) and the decreases in neighborhood safety (negative influences).

Furthermore, in our study, only right, but not left, insula and ACC reactivity to emotion moderated the association between changes in neighborhood safety and adolescent adjustment. Such findings provide support for the right-hemispheric dominance hypothesis about emotional processing (Borod et al., 1998, Gainotti, 2019). Specifically, this theory hypothesizes that all emotions are processed in the right hemisphere, independent of their valence or of the emotional feeling being processed. Empirical studies also support right insula as the major hub for visceral responses accessible to awareness and the emergence of subjective feeling states (Critchley et al., 2004). Indeed, specific roles of each hemisphere in emotional processing still remain an issue of controversy, reflected in the continued debate in recent reviews (Gainotti, 2022, Palomero-Gallagher and Amunts, 2022). It is important for future studies to further investigate if the right hemisphere, specifically right insula and ACC, processes the emotional cues in individuals’ surrounding environment.

However, we did not find evidence that amygdala reactivity moderates the association between changes in neighborhood safety and adolescent adjustment. One possible explanation is that, compared with the insula which plays a greater role in integrating affective and cognitive processes, the amygdala may have a more selective role in affective arousal, especially for negative stimuli (Berntson et al., 2011). Perception of safety cues in the neighborhood may be more related to the combination of affective and cognitive processes rather than only affective arousal. It is also possible that amygdala reactivity may be a marker of differential susceptibility in older adolescents or adults as compared to early adolescents (ages 9–10 at baseline) in the current study (Gard et al., 2018).

Also, neural reactivity to negative emotion did not moderate the association between changes in neighborhood safety and adolescent adjustment. One possible explanation is that, rather than serving as a potential moderator, adolescents’ neural reactivity to negative emotional stimuli (i.e., fear) may be directly influenced by threats in the neighborhood context. Previous studies found that neighborhood disadvantage was linked with blunted physiological (e.g., HPA axis; Busso et al., 2017) and neural reactivity (e.g., amygdala; Huggins et al., 2022) to stress and threat during early adolescence. Future studies can further explore the link between neighborhood safety and longitudinal changes in adolescents’ brain function to better understand if brain functions serve as a potential mechanism linking these associations.

Additionally, no moderation was found for the link between changes in neighborhood safety and externalizing symptoms, suggesting that the negative influence of decreases in neighborhood safety on externalizing symptoms is universal to youth during the transitional period of early adolescence, rather than vary by individual characteristics. It is also possible that other brain regions, rather than insula and ACC, may play a role in the development of externalizing symptoms. For example, a recent study found posterior cingulate, temporoparietal junction, and amygdala reaction during sadness introspection moderated the association between community crime exposure and externalizing symptoms among a group of Mexican-origin adolescents (Weissman et al., 2018).

Notable strengths of this study include utilizing large-scale national longitudinal data of adolescents, focusing on the effects of both baseline level and changes in neighborhood safety on adolescent adjustment, and identifying neural susceptibility factors for the link between changes in neighborhood safety and adolescent adjustment. This study also has limitations. First, this study only focused on parent-reported neighborhood safety and parent-reported adolescent adjustment. Although the ABCD study also includes youth-reported neighborhood safety, the construct is measured based on a single item, of which the reliability cannot be assessed. Also, the ABCD study does not have youth-reported internalizing symptoms, externalizing symptoms, and sleep disturbance at baseline. Future studies may use adolescents’ own reports as well as objective indicators of neighborhood safety and adjustment outcomes to further validate the findings of our study. Second, participants on average reported relatively high neighborhood safety across the three waves of data collection. Although participants in the ABCD dataset reported the full range of neighborhood safety, it is unclear if a study with a higher percentage of adolescents living in neighborhoods with lower safety may confer different patterns of findings, or further validate the differential susceptibility hypothesis. Thus, future studies with adolescents in disadvantaged neighborhoods are needed to further understand whether adolescents’ affective neural sensitivity moderates the link between changes in neighborhood safety and adolescent adjustment. Third, this study only focused on one aspect of neighborhood characteristics (i.e., safety). Future studies could examine other aspects of the neighborhood environment, including negative characteristics such as deprivation and positive aspects such as neighborhood opportunities. Fourth, the current study focused on adolescents’ neural activation to two emotional stimuli: happy and fearful faces. Future studies can examine adolescents’ neural responses to other emotional stimuli, such as angry and sad faces, to better understand how neural responses to different emotions may influence how adolescents perceive cues in the surrounding environment. Moreover, other neurobiological factors could be probed in further research, including the functional connectivity of the salience network that contains both insula and ACC (Seeley, 2019). Fifth, this study used emotion contrasts from emotional n-back task, using the processed neuroimaging data available at the ABCD 4.0 release. Yet, such contrasts did not distinguish the task conditions with (i.e., 2-back) and without (i.e., 0-back) a cognitive load. Future studies should separate the conditions with and without cognitive load, to provide a better understanding of how neural reactivity to emotion serves as a factor of differential susceptibility. Finally, the present study focused on adolescents’ adjustment problems, especially internalizing symptoms, externalizing symptoms, and sleep disturbance. Indeed, the lack of mental and physical health symptoms does not necessarily represent other dimensions of positive adjustment outcomes, such as prosocial behaviors. Future studies should consider investigating adolescents’ positive outcomes to have a more holistic view of adolescent adjustment.

In sum, our findings demonstrate the importance of neighborhood safety in adolescent adjustment, signifying that both higher baseline levels and increases in neighborhood safety can improve adolescents’ development. In addition, our findings also highlight higher neural reactivity in right insula and ACC to positive emotion as susceptibility factors within the context of a changing neighborhood environment. The findings indicate that improving neighborhood safety can be beneficial during the transition to adolescence and that understanding adolescents’ neural characteristics can help to develop targeted interventions to facilitate positive youth development.

## CRediT authorship contribution statement

**Claudia M. Haase:** Writing – review & editing, Data curation. **Emma K Adam:** Writing – review & editing. **Yang Qu:** Writing – review & editing, Supervision, Investigation, Data curation. **Ka I Ip:** Writing – review & editing. **Zexi Zhou:** Writing – review & editing. **Beiming Yang:** Writing – review & editing, Writing – original draft, Data curation. **Tianying Cai:** Writing – review & editing, Writing – original draft, Visualization, Software, Methodology, Investigation, Formal analysis, Data curation, Conceptualization.

## Declaration of Competing Interest

None.

## Data Availability

Data were obtained from baseline, one-year follow up and two-year follo-up of the Adolescent Brain Cognitive Development (ABCD) study (data release 4.0). All the data included in the current study are availablle on the NIMH Data Archive (https://nda.nih.gov/abcd) upon data access request.
