# Supplementary material for: Longitudinal associations between neighborhood safety and adolescent adjustment: The moderating role of affective neural sensitivity
Source: Dev Cogn Neurosci. 2024 Apr 12;67:101380. doi: 10.1016/j.dcn.2024.101380 (PMC11035046; doi:10.1016/j.dcn.2024.101380)
Supplement: Supplementary file 1 — Supplementary material [file mmc1.docx]

Supplementary Figure 1

*Latent Growth Curve Models of Neighborhood Safety Predicting Externalizing Symptoms, Internalizing Symptoms, and Sleep Disturbance*

*Note*: Standardized estimations were presented.

^*^ *p* < .05. ^**^ *p* < .01. ^***^ *p* < .001.
