# Supplementary material for: Longitudinal associations between neighborhood safety and adolescent adjustment: The moderating role of affective neural sensitivity
Source: Dev Cogn Neurosci. 2024 Apr 12;67:101380. doi: 10.1016/j.dcn.2024.101380 (PMC11035046; doi:10.1016/j.dcn.2024.101380)
Supplement: Supplementary file 2 — Supplementary material [file mmc2.docx]

Supplementary Table 1

*Interaction between Changes in Neighborhood Safety and Neural Reactivity to Positive Emotion Predicting Parent-Reported Adolescent Adjustment*

|  | **Externalizing Symptoms (CBCL)** | | | |  |  | **Internalizing Symptoms (CBCL)** | | | |  |  | **Sleep Disturbance** | | | |  |
| --- | --- | --- | --- | --- | --- | --- | --- | --- | --- | --- | --- | --- | --- | --- | --- | --- | --- |
|  | *B* | *SE* | β | *p^uc^* | *p^c^* |  | *B* | *SE* | β | *p^uc^* | *p^c^* |  | *B* | *SE* | β | *p^uc^* | *p^c^* |
| ***Right Insula Reactivity to Positive Emotion*** |  |  |  |  |  |  |  |  |  |  |  |  |  |  |  |  |  |
| *NS*_change_ | -7.34 | 2.11 | -.14 | .000 | .000 |  | -4.66 | 1.82 | -.08 | .010 | .037 |  | -2.54 | 1.38 | -.06 | .066 | .137 |
| Right Insula Reactivity | -.65 | .27 | -.02 | .018 | .022 |  | -.45 | .33 | -.02 | .167 | .167 |  | -.03 | .21 | .00 | .875 | .978 |
| *NS*_change_ x Right Insula Reactivity | -6.61 | 3.73 | -.04 | .076 | .338 |  | **-10.71** | **4.42** | **-.06** | **.015** | **.045** |  | **-7.39** | **2.68** | **-.06** | **.006** | **.036** |
| ***Right ACC Reactivity to Positive Emotion*** |  |  |  |  |  |  |  |  |  |  |  |  |  |  |  |  |  |
| *NS*_change_ | -9.43 | 3.30 | -.16 | .004 | .008 |  | -6.48 | 2.74 | -.10 | .018 | .037 |  | -2.82 | 1.78 | -.06 | .114 | .137 |
| Right ACC Reactivity | -.68 | .20 | -.03 | .001 | .006 |  | -.77 | .30 | -.03 | .011 | .066 |  | -.28 | .17 | -.02 | .094 | .426 |
| *NS*_change_ x Right ACC Reactivity | -4.83 | 3.55 | -.04 | .174 | .338 |  | **-13.47** | **5.42** | **-.09** | **.013** | **.045** |  | -2.95 | 2.69 | -.03 | .272 | .272 |
| ***Right Amygdala Reactivity to Positive Emotion*** |  |  |  |  |  |  |  |  |  |  |  |  |  |  |  |  |  |
| *NS*_change_ | -8.27 | 2.71 | -.15 | .002 | .006 |  | -4.92 | 2.18 | -.08 | .024 | .037 |  | -2.76 | 1.65 | -.06 | .094 | .137 |
| Right Amygdala Reactivity | -.52 | .20 | -.03 | .008 | .012 |  | -.34 | .19 | -.02 | .077 | .100 |  | -.19 | .15 | -.01 | .213 | .426 |
| *NS*_change_ x Right Amygdala Reactivity | 4.87 | 3.60 | .04 | .176 | .338 |  | -1.52 | 2.55 | -.01 | .550 | .660 |  | -5.02 | 2.83 | -.05 | .076 | .228 |
| ***Left Insula Reactivity to Positive Emotion*** |  |  |  |  |  |  |  |  |  |  |  |  |  |  |  |  |  |
| *NS*_change_ | -8.21 | 2.93 | -.15 | .005 | .008 |  | -5.12 | 2.28 | -.08 | .025 | .037 |  | -2.72 | 1.64 | -.06 | .097 | .137 |
| Left Insula Reactivity | -.77 | 0.27 | -.03 | .005 | .010 |  | -.67 | .34 | -.02 | .047 | .094 |  | -.31 | .23 | -.01 | .170 | .426 |
| *NS*_change_ x Left Insula Reactivity | -3.90 | 3.22 | -.02 | .225 | .338 |  | -6.29 | 4.42 | -.03 | .155 | .290 |  | -4.14 | 2.93 | -.03 | .158 | .272 |
| ***Left ACC Reactivity to Positive Emotion*** |  |  |  |  |  |  |  |  |  |  |  |  |  |  |  |  |  |
| *NS*_change_ | -9.25 | 3.61 | -.16 | .010 | .012 |  | -6.22 | 2.88 | -.10 | .031 | .037 |  | -3.11 | 1.92 | -.06 | .105 | .137 |
| Left ACC Reactivity | -.58 | .20 | -.03 | .004 | .010 |  | -.60 | .27 | -.03 | .023 | .069 |  | -.15 | 0.18 | -.01 | .407 | .610 |
| *NS*_change_ x Left ACC Reactivity | -1.17 | 2.96 | -.01 | .694 | .724 |  | -7.28 | 5.57 | -.05 | .191 | .290 |  | -3.58 | 2.79 | -.03 | .199 | .272 |
| ***Left Amygdala Reactivity to Positive Emotion*** |  |  |  |  |  |  |  |  |  |  |  |  |  |  |  |  |  |
| *NS*_change_ | -8.83 | 4.02 | -.15 | .028 | .028 |  | -5.31 | 2.99 | -.08 | .076 | .076 |  | -3.05 | 2.09 | -.06 | .144 | .144 |
| Left Amygdala Reactivity | -.32 | .22 | -.02 | .153 | .015 |  | -0.45 | .26 | -.02 | .083 | .100 |  | .00 | .16 | .00 | .978 | .978 |
| *NS*_change_ x Left Amygdala Reactivity | 2.21 | 6.26 | .02 | .724 | .724 |  | .07 | 8.06 | .00 | .993 | .993 |  | -2.72 | 2.30 | -.03 | .238 | .272 |

*Note.* Significant interactions are highlighted after correction for multiple testing using the FDR method. All three adolescent adjustment outcomes were tested in one model, resulting in a total of 6 interaction models (right insula, right ACC, right amygdala, left insula, left ACC, left right amygdala). *p^uc^* represents unadjusted p-values.  *p^c^* represents FDR adjusted *p*-values for the 6 models. CBCL = Child Behavior Checklist.

Supplementary Table 2

*Interaction between Changes in Neighborhood Safety and Neural Reactivity to Negative Emotion Predicting Parent-Reported Adolescent Adjustment*

|  | **Externalizing Symptoms (CBCL)** | | | |  |  | **Internalizing Symptoms (CBCL)** | | | |  |  | **Sleep Disturbance** | | | |  |
| --- | --- | --- | --- | --- | --- | --- | --- | --- | --- | --- | --- | --- | --- | --- | --- | --- | --- |
|  | *B* | *SE* | β | *p^uc^* | *p^c^* |  | *B* | *SE* | β | *p^uc^* | *p^c^* |  | *B* | *SE* | β | *p^uc^* | *p^c^* |
| ***Right Insula Reactivity to Negative Emotion*** |  |  |  |  |  |  |  |  |  |  |  |  |  |  |  |  |  |
| *NS*_change_ | -9.21 | 5.12 | -.16 | .072 | .077 |  | -5.63 | 3.09 | -.09 | .068 | .068 |  | -3.41 | 3.64 | -.07 | .349 | .349 |
| Right Insula Reactivity | -.32 | .47 | -.01 | .497 | .589 |  | -.07 | .47 | .00 | .875 | .985 |  | -.08 | .34 | .00 | .811 | .811 |
| *NS*_change_ x Right Insula Reactivity | 3.03 | 14.01 | .02 | .829 | .829 |  | 3.82 | 13.96 | .02 | .784 | .784 |  | 4.15 | 14.77 | .03 | .779 | .880 |
| ***Right ACC Reactivity to Negative Emotion*** |  |  |  |  |  |  |  |  |  |  |  |  |  |  |  |  |  |
| *NS*_change_ | -8.69 | 3.18 | -.15 | .006 | .027 |  | -5.60 | 2.55 | -.09 | .028 | .068 |  | -2.67 | 2.24 | -.06 | .232 | .348 |
| Right ACC Reactivity | -.77 | .23 | -.04 | .001 | .006 |  | -.78 | .32 | -.03 | .014 | .084 |  | -.37 | .21 | -.02 | .081 | .486 |
| *NS*_change_ x Right ACC Reactivity | 1.90 | 3.50 | .01 | .587 | .704 |  | -7.08 | 6.47 | -.05 | .274 | .411 |  | -.77 | 5.11 | -.01 | .880 | .880 |
| ***Right Amygdala Reactivity to Negative Emotion*** | |  |  |  |  |  |  |  |  |  |  |  |  |  |  |  |  |
| *NS*_change_ | -10.64 | 6.02 | -.17 | .077 | .077 |  | -5.76 | 3.05 | -.08 | .059 | .068 |  | -2.93 | 2.09 | -.06 | .161 | .348 |
| Right Amygdala Reactivity | -.25 | .25 | -.01 | .316 | .589 |  | .20 | .24 | .01 | .410 | .820 |  | -.16 | .16 | -.01 | .308 | .599 |
| *NS*_change_ x Right Amygdala Reactivity | 6.73 | 5.82 | .05 | .247 | .669 |  | 5.59 | 4.64 | .04 | .229 | .411 |  | -1.37 | 2.54 | -.01 | .588 | .880 |
| ***Left Insula Reactivity to Negative Emotion*** |  |  |  |  |  |  |  |  |  |  |  |  |  |  |  |  |  |
| *NS*_change_ | -10.83 | 4.79 | -.17 | .024 | .048 |  | -6.66 | 3.36 | -.09 | .047 | .068 |  | -4.27 | 3.57 | -.08 | .231 | .348 |
| Left Insula Reactivity | -.22 | .39 | -.01 | .570 | .589 |  | .01 | .40 | .00 | .985 | .985 |  | -.19 | .27 | -.01 | .499 | .599 |
| *NS*_change_ x Left Insula Reactivity | 14.02 | 9.48 | .07 | .139 | .669 |  | 16.78 | 8.85 | .08 | .058 | .348 |  | 9.74 | 10.86 | .06 | .370 | .880 |
| ***Left ACC Reactivity to Negative Emotion*** |  |  |  |  |  |  |  |  |  |  |  |  |  |  |  |  |  |
| *NS*_change_ | -9.84 | 4.66 | -.16 | .035 | .053 |  | -6.18 | 3.36 | -.09 | .066 | .068 |  | -3.50 | 3.35 | -.07 | .296 | .349 |
| Left ACC Reactivity | -.50 | .24 | -.02 | .034 | .102 |  | -.33 | .35 | -.01 | .355 | .820 |  | -.16 | .23 | -.01 | .487 | .599 |
| *NS*_change_ x Left ACC Reactivity | 3.83 | 4.25 | .03 | .367 | .669 |  | -3.34 | 8.37 | -.02 | .690 | .784 |  | 1.97 | 7.86 | .02 | .802 | .880 |
| ***Left Amygdala Reactivity to Negative Emotion*** |  |  |  |  |  |  |  |  |  |  |  |  |  |  |  |  |  |
| *NS*_change_ | -8.83 | 3.39 | -.15 | .009 | .027 |  | -5.59 | 2.57 | -.09 | .030 | .068 |  | -3.09 | 2.01 | -.07 | .123 | .348 |
| Left Amygdala Reactivity | -.11 | .20 | -.01 | .589 | .589 |  | .05 | .23 | .00 | .836 | .985 |  | -.16 | .15 | -.01 | .274 | .599 |
| *NS*_change_ x Left Amygdala Reactivity | 1.96 | 2.57 | 0.02 | .446 | .669 |  | 3.63 | 3.17 | .03 | .252 | .411 |  | 1.48 | 2.61 | .02 | .569 | .880 |

*Note.* All three adolescent adjustment outcomes were tested in one model, resulting in a total of 6 interaction models (right insula, right ACC, right amygdala, left insula, left ACC, left amygdala). *p^uc^* represents unadjusted p-values.  *p^c^* represents FDR adjusted *p*-values for the 6 models. CBCL = Child Behavior Checklist.

***Sensitivity Analyses Set 1: Sub-Regions of Insula***

Supplementary Table 3

*Sensitivity Analyses: Interaction between Changes in Neighborhood Safety and Right Insula Sub-Region Reactivity to Positive Emotion Predicting Adolescent Adjustment*

|  | **Externalizing Symptoms** | | | |  | **Internalizing Symptoms** | | | |  | **Sleep Disturbance** | | | |
| --- | --- | --- | --- | --- | --- | --- | --- | --- | --- | --- | --- | --- | --- | --- |
|  | *B* | *SE* | β | *p* |  | *B* | *SE* | β | *p* |  | *B* | *SE* | β | *p* |
| ***Right Anterior Insula Reactivity to Positive Emotion*** |  |  |  |  |  |  |  |  |  |  |  |  |  |  |
| *NS*_change_ | -8.23 | 2.60 | -.15 | .002 |  | -5.17 | 2.10 | -.09 | .014 |  | -2.73 | 1.54 | -.06 | .076 |
| Right Anterior Insula Reactivity | -.61 | .21 | -.03 | .004 |  | -.69 | .26 | -.03 | .008 |  | -.20 | .16 | -.01 | .228 |
| *NS*_change_ x Right Anterior Insula Reactivity | -4.25 | 4.04 | -.03 | .292 |  | **-8.48** | **3.90** | **-.06** | **.030** |  | **-3.82** | **1.92** | **-.04** | **.047** |
| ***Right Inferior Insula Reactivity to Positive Emotion*** |  |  |  |  |  |  |  |  |  |  |  |  |  |  |
| *NS*_change_ | -8.00 | 2.38 | -.14 | .001 |  | -4.59 | 1.95 | -.08 | .019 |  | -2.59 | 1.55 | -.06 | .095 |
| Right Inferior Insula Reactivity | -.48 | .26 | -.02 | .065 |  | -.52 | .33 | -.02 | .116 |  | .08 | .20 | .00 | .696 |
| *NS*_change_ x Right Inferior Insula Reactivity | -6.88 | 3.71 | -.05 | .064 |  | **-11.39** | **5.42** | **-.07** | **.036** |  | -4.75 | 2.78 | -.04 | .087 |
| ***Right Superior Insula Reactivity*** |  |  |  |  |  |  |  |  |  |  |  |  |  |  |
| *NS*_change_ | -8.54 | 2.99 | -.15 | .004 |  | -5.49 | 2.42 | -.09 | .024 |  | -3.02 | 1.74 | -.06 | .083 |
| Right Superior Insula Reactivity | -.60 | .24 | -.03 | .012 |  | -.29 | .28 | -.01 | .312 |  | -0.06 | .19 | .00 | .763 |
| *NS*_change_ x Right Superior Insula Reactivity | -5.37 | 3.20 | -.04 | .093 |  | -7.41 | 4.11 | -.05 | .072 |  | -5.39 | 3.04 | -.04 | .077 |
| ***Right Long Insular Gurus and Central Sulcus***  ***Reactivity to Positive Emotion*** |  |  |  |  |  |  |  |  |  |  |  |  |  |  |
| *NS*_change_ | -8.35 | 2.62 | -.15 | .001 |  | -5.08 | 2.11 | -.08 | .016 |  | -2.30 | 1.52 | -.05 | .130 |
| Right Long Insular Gurus and Central Sulcus  Reactivity | -.49 | .24 | -.02 | .043 |  | -.32 | .28 | -.01 | .257 |  | .02 | .19 | .00 | .919 |
| *NS*_change_ x Right Long Insular Gurus and Central Sulcus  Reactivity | -6.74 | 4.59 | -.05 | .142 |  | **-8.61** | **4.19** | **-.06** | **.040** |  | **-6.94** | **3.12** | **-.06** | **.026** |
| ***Right Short Insular Gyri***  ***Reactivity to Positive Emotion*** |  |  |  |  |  |  |  |  |  |  |  |  |  |  |
| *NS*_change_ | -7.35 | 2.30 | -.14 | .001 |  | -4.57 | 1.92 | -.08 | .017 |  | -2.31 | 1.41 | -.05 | .101 |
| Right Short Insular Gyri Reactivity | -.40 | .21 | -.02 | .055 |  | -.26 | .25 | -.01 | .298 |  | -.06 | .18 | -.00 | .751 |
| *NS*_change_ x Right Short Insular Gyri Reactivity | -3.18 | 2.22 | -.03 | .152 |  | **-6.59** | **2.98** | **-.05** | **.027** |  | **-4.70** | **2.29** | **-.04** | **.040** |

*Note*. All three adolescent adjustment outcomes were tested in one model, resulting in a total of 5 models representing right insula sub-region reactivity to positive emotion.

***Sensitivity Analyses Set 2: Extreme Values of Neural Reactivity***

Supplementary Table 4

*Sensitivity Analyses: Interaction between Changes in Neighborhood Safety and Neural Reactivity to Positive Emotion (Outlier Trimmed to +/- 3 SD) Predicting Adolescent Adjustment*

|  | **Externalizing Symptoms** | | | |  | **Internalizing Symptoms** | | | |  | **Sleep Disturbance** | | | | |
| --- | --- | --- | --- | --- | --- | --- | --- | --- | --- | --- | --- | --- | --- | --- | --- |
|  | *B* | *SE* | β | *p* |  | *B* | *SE* | β | *p* |  | *B* | *SE* | β | *p* |  |
| ***Right Insula Reactivity to Positive Emotion*** |  |  |  |  |  |  |  |  |  |  |  |  |  |  |  |
| *NS*_change_ | -7.83 | 2.26 | -.14 | .001 |  | -4.86 | 1.93 | -.08 | .012 |  | -2.67 | 1.49 | -.06 | .074 |  |
| Right Insula Reactivity | -.77 | 0.34 | -.03 | .022 |  | -.58 | .38 | -.02 | .128 |  | -.09 | .26 | .00 | .739 |  |
| *NS*_change_ x Right Insula Reactivity | -8.89 | 4.77 | -.05 | .062 |  | **-13.52** | **5.30** | **-.07** | **.011** |  | **-10.12** | **4.05** | **-.07** | **.012** |  |
| ***Right ACC Reactivity to Positive Emotion*** |  |  |  |  |  |  |  |  |  |  |  |  |  |  |  |
| *NS*_change_ | -9.04 | 3.04 | -.15 | .003 |  | -6.30 | 2.58 | -.10 | .014 |  | -2.74 | 1.70 | -.06 | .108 |  |
| Right ACC Reactivity | -.80 | .26 | -.03 | .003 |  | -.85 | .33 | -.03 | .010 |  | -.21 | .21 | -.01 | .308 |  |
| *NS*_change_ x Right ACC Reactivity | -5.66 | 4.23 | -.04 | .180 |  | **-15.69** | **6.08** | **-.09** | **.010** |  | -3.00 | 3.29 | -.02 | .363 |  |
| ***Right Amygdala Reactivity to Positive Emotion*** |  |  |  |  |  |  |  |  |  |  |  |  |  |  |  |
| *NS*_change_ | -8.19 | 2.56 | -.15 | .001 |  | -4.80 | 2.12 | -.08 | .023 |  | -2.64 | 1.61 | -.06 | .100 |  |
| Right Amygdala Reactivity | -.47 | .25 | -.02 | .056 |  | -.25 | .28 | -.01 | .372 |  | -.22 | .20 | -.01 | .265 |  |
| *NS*_change_ x Right Amygdala Reactivity | 5.60 | 3.70 | .04 | .130 |  | -2.06 | 4.23 | -.01 | .626 |  | -6.90 | 3.85 | -.06 | .073 |  |
| ***Left Insula Reactivity to Positive Emotion*** |  |  |  |  |  |  |  |  |  |  |  |  |  |  |  |
| *NS*_change_ | -8.11 | 3.01 | -.15 | .007 |  | -5.05 | 2.29 | -.08 | .027 |  | -2.67 | 1.63 | -.06 | .100 |  |
| Left Insula Reactivity | -.81 | .33 | -.03 | .014 |  | -.74 | .40 | -.02 | .061 |  | -.37 | .26 | -.01 | .162 |  |
| *NS*_change_ x Left Insula Reactivity | -3.97 | 4.85 | -.02 | .414 |  | -7.10 | 5.89 | -.03 | .228 |  | -4.87 | 4.13 | -.03 | .238 |  |
| ***Left ACC Reactivity to Positive Emotion*** |  |  |  |  |  |  |  |  |  |  |  |  |  |  |  |
| *NS*_change_ | -8.93 | 3.33 | -.15 | .007 |  | -6.12 | 2.71 | -.10 | .024 |  | -2.95 | 1.81 | -.06 | .102 |  |
| Left ACC Reactivity | -.56 | .26 | -.02 | .033 |  | -.57 | .33 | -.02 | .082 |  | -.07 | .21 | .00 | .753 |  |
| *NS*_change_ x Left ACC Reactivity | -1.24 | 4.22 | -.01 | .770 |  | -9.07 | 5.94 | -.05 | .127 |  | -2.88 | 3.32 | -.02 | .387 |  |
| ***Left Amygdala Reactivity to Negative Emotion*** |  |  |  |  |  |  |  |  |  |  |  |  |  |  |  |
| *NS*_change_ | -9.58 | 4.94 | -.16 | .053 |  | -5.74 | 3.30 | -.09 | .082 |  | -3.37 | 2.51 | -.07 | .181 |  |
| Left Amygdala Reactivity | -.26 | .28 | -.01 | .353 |  | -.49 | .32 | -.02 | .121 |  | .02 | .19 | .00 | .920 |  |
| *NS*_change_ x Left Amygdala Reactivity | 3.95 | 7.49 | .03 | .598 |  | .92 | 7.58 | .01 | .903 |  | -3.69 | 3.54 | -.03 | .297 |  |

*Note*. All three adolescent adjustment outcomes were tested in one model, resulting in a total of 6 interaction models (right insula, right ACC, right amygdala, left insula, left ACC, left right amygdala).

***Sensitivity Analyses Set 3: Youth Report of Externalizing/Internalizing Symptoms via Brief Problem Monitor***

This set of sensitivity analyses examined the role of neighborhood safety in adolescent adjustment and the moderating role of neural reactivity to emotional stimuli with youth reports of externalizing and internalizing symptoms, measured by the Brief Problem Monitor (BPM). It is important to note that the analyses are fundamentally different. First, given that the BPM was not included in the baseline assessment, we were not able to control for baseline youth-reported symptoms in the models. This is different from stringent analyses with parent reports of adolescents’ symptoms via CBCL, in which we controlled for adolescents’ externalizing/internalizing symptoms at baseline. Second, youth reports and parent reports of adolescents’ symptoms were only weakly correlated (*r* = .31 for externalizing symptoms, *r* = .27 for internalizing symptoms). The BPM has much fewer items than the CBCL and the conception of the symptoms is simplified (CBCL: 35-item externalizing and 32-item internalizing symptoms; BPM: 7-item externalizing and 6-item internalizing symptoms). Third, whereas the CBCL has high reliability, the BPM measure has relatively low reliability (e.g., α = .67 for externalizing symptoms).

Supplementary Table 5

*Sensitivity Analyses: Latent Growth Curve Model of Neighborhood Safety Predicting Adolescent Adjustment*

|  | **Externalizing Symptoms**  **(BPM)** | | |  | **Internalizing Symptoms**  **(BPM)** | | |  |
| --- | --- | --- | --- | --- | --- | --- | --- | --- |
|  | *B* | *SE* | β |  | *B* | *SE* | β |  |
| **Predictor** |  |  |  |  |  |  |  |  |
| Intercept (Initial Level of Neighborhood Safety) | -.14 | .09 | -.03 |  | -.10 | .11 | -.01 |  |
| Slope (Changes of Neighborhood Safety) | -1.90 | 1.02 | -.08 |  | -.50 | 1.04 | -.02 |  |
| ***Covariates*** |  |  |  |  |  |  |  |  |
| Adolescent Adjustment (6-month follow-up) | .39*** | .02 | .37 |  | .39*** | .02 | .36 |  |
| Race-Black | .13 | .20 | .01 |  | -.72** | .23 | -.04 |  |
| Race-Latino | .42** | .14 | .04 |  | .56** | .19 | .04 |  |
| Race-Asian | .12 | .26 | .00 |  | -.56 | .33 | -.02 |  |
| Race-Other | .24 | .16 | .02 |  | .51* | .23 | .03 |  |
| Age | .02*** | .01 | .04 |  | .03*** | .01 | .04 |  |
| Sex at birth | .30** | .09 | .04 |  | .67*** | .12 | .06 |  |
| Parental Educational Attainment | -.03 | .05 | -.01 |  | .00 | .07 | .00 |  |
| ADI | .01** | .00 | .05 |  | .00 | .00 | .01 |  |
| Family Conflict | .22*** | .03 | .11 |  | .12*** | .03 | .04 |  |
|  |  |  |  |  |  |  |  |  |
| ***Model Fit*** |  |  |  |  |  |  |  |  |
| RMSEA | .019 |  |  |  |  |  |  |  |
| CFI | .996 |  |  |  |  |  |  |  |
| TLI | .979 |  |  |  |  |  |  |  |
| SRMR | .018 |  |  |  |  |  |  |  |

*Note*: Youth-reported externalizing and internalizing symptoms were measured by Brief Problem Monitor (BPM). Both outcomes were included in one model. ^*^ *p* < .05. ^**^ *p* < .01. ^***^ *p* < .001.

Supplementary Table 6

*Sensitivity Analyses: Interaction between Changes in Neighborhood Safety and Neural Reactivity to Positive Emotion Predicting Youth-Reported Adolescent Adjustment*

|  | **Externalizing Symptoms**  **(BPM)** | | | |  |  | **Internalizing Symptoms**  **(BPM)** | | | |  |
| --- | --- | --- | --- | --- | --- | --- | --- | --- | --- | --- | --- |
|  | *B* | *SE* | β | *p^uc^* | *p^c^* |  | *B* | *SE* | β | *p^uc^* | *p^c^* |
| ***Right Insula Reactivity to Positive Emotion*** |  |  |  |  |  |  |  |  |  |  |  |
| *NS*_change_ | -1.67 | .86 | -.07 | .052 | .100 |  | -.47 | .93 | -.02 | .612 | .777 |
| Right Insula Reactivity | -.25 | .16 | -.02 | .112 | .655 |  | -.08 | .22 | -.01 | .730 | .730 |
| *NS*_change_ x Right Insula Reactivity | -1.14 | 4.07 | -.02 | .780 | .961 |  | -1.67 | 4.32 | -.02 | .699 | .935 |
| ***Right ACC Reactivity to Positive Emotion*** |  |  |  |  |  |  |  |  |  |  |  |
| *NS*_change_ | -1.59 | .87 | -.07 | .066 | .100 |  | -.44 | .91 | -.01 | .632 | .777 |
| Right ACC Reactivity | -.07 | .12 | -.01 | .591 | .709 |  | -.12 | .19 | -.01 | .531 | .637 |
| *NS*_change_ x Right ACC Reactivity | -.16 | 3.28 | .00 | .961 | .961 |  | -1.63 | 3.13 | -.02 | .602 | .935 |
| ***Right Amygdala Reactivity to Positive Emotion*** |  |  |  |  |  |  |  |  |  |  |  |
| *NS*_change_ | -1.62 | .88 | -.07 | .067 | .100 |  | -.37 | .96 | -.01 | .701 | .777 |
| Right Amygdala Reactivity | .07 | .10 | .01 | .437 | .655 |  | .15 | .13 | .01 | .263 | .637 |
| *NS*_change_ x Right Amygdala Reactivity | .86 | 2.46 | .02 | .726 | .961 |  | -.36 | 2.38 | -.01 | .881 | .935 |
| ***Left Insula Reactivity to Positive Emotion*** |  |  |  |  |  |  |  |  |  |  |  |
| *NS*_change_ | -1.75 | 1.02 | -.07 | .085 | .102 |  | -.48 | 1.33 | -.01 | .716 | .777 |
| Left Insula Reactivity | -.39 | .47 | -.03 | .403 | .655 |  | -.34 | .22 | -.02 | .127 | .637 |
| *NS*_change_ x Left Insula Reactivity | -4.50 | 17.08 | -.06 | .792 | .961 |  | -1.28 | 4.60 | -.01 | .781 | .935 |
| ***Left ACC Reactivity to Positive Emotion*** |  |  |  |  |  |  |  |  |  |  |  |
| *NS*_change_ | -1.67 | .88 | -.07 | .057 | .100 |  | -.50 | .91 | -.02 | .585 | .777 |
| Left ACC Reactivity | -.14 | .11 | -.01 | .231 | .655 |  | -.15 | .17 | -.01 | .393 | .637 |
| *NS*_change_ x Left ACC Reactivity | -.62 | 2.79 | -.01 | .823 | .961 |  | -3.04 | 3.02 | -.04 | .315 | .935 |
| ***Left Amygdala Reactivity to Positive Emotion*** |  |  |  |  |  |  |  |  |  |  |  |
| *NS*_change_ | -2.12 | 2.49 | -.08 | .395 | .395 |  | -.37 | 1.31 | -.01 | .777 | .777 |
| Left Amygdala Reactivity | .03 | .23 | .00 | .886 | .886 |  | .12 | .17 | .01 | .469 | .637 |
| *NS*_change_ x Left Amygdala Reactivity | 4.78 | 14.90 | .08 | .748 | .961 |  | -.55 | 6.80 | -.01 | .935 | .935 |

*Note.* Youth-reported externalizing and internalizing symptoms were measured by Brief Problem Monitor (BPM). Both adolescent adjustment outcomes were tested in one model, resulting in a total of 6 interaction models (right insula, right ACC, right amygdala, left insula, left ACC, left right amygdala). *p^uc^* represents unadjusted p-values.  *p^c^* represents FDR adjusted *p*-values for the 6 models.

Supplementary Table 7

*Sensitivity Analyses: Interaction between Changes in Neighborhood Safety and Neural Reactivity to Negative Emotion Predicting Youth-Reported Adolescent Adjustment*

|  | **Externalizing Symptoms**  **(BPM)** | | | |  |  | **Internalizing Symptoms**  **(BPM)** | | | |  |
| --- | --- | --- | --- | --- | --- | --- | --- | --- | --- | --- | --- |
|  | *B* | *SE* | β | *p^uc^* | *p^c^* |  | *B* | *SE* | β | *p^uc^* | *p^c^* |
| ***Right Insula Reactivity to Negative Emotion*** |  |  |  |  |  |  |  |  |  |  |  |
| *NS*_change_ | -2.52 | 2.26 | -.10 | .264 | .471 |  | -.94 | 1.63 | -.03 | .563 | .859 |
| Right Insula Reactivity | -.04 | .19 | .00 | .835 | .963 |  | -.04 | .19 | .00 | .829 | .882 |
| *NS*_change_ x Right Insula Reactivity | 6.35 | 7.02 | .08 | .366 | .764 |  | 1.81 | 3.72 | .02 | .626 | .699 |
| ***Right ACC Reactivity to Negative Emotion*** |  |  |  |  |  |  |  |  |  |  |  |
| *NS*_change_ | -3.17 | 6.58 | -.11 | .630 | .638 |  | .01 | .20 | -.06 | .962 | .962 |
| Right ACC Reactivity | .01 | .29 | .00 | .963 | .963 |  | -2.13 | 6.35 | .00 | .737 | .882 |
| *NS*_change_ x Right ACC Reactivity | 6.32 | 13.39 | .09 | .637 | .764 |  | -0.10 | .11 | .00 | .371 | .699 |
| ***Right Amygdala Reactivity to Negative Emotion*** |  |  |  |  |  |  |  |  |  |  |  |
| *NS*_change_ | -1.70 | 0.83 | -.07 | .040 | .156 |  | -0.56 | .97 | -.02 | .565 | .859 |
| Right Amygdala Reactivity | .17 | .12 | .02 | .157 | .471 |  | .24 | .15 | .02 | .111 | .666 |
| *NS*_change_ x Right Amygdala Reactivity | 2.52 | 3.20 | 0.05 | .431 | .764 |  | -1.55 | 3.84 | -.02 | .687 | .699 |
| ***Left Insula Reactivity to Negative Emotion*** |  |  |  |  |  |  |  |  |  |  |  |
| *NS*_change_ | -11.53 | 24.54 | -.21 | .638 | .638 |  | -6.98 | 19.18 | -.10 | .716 | .859 |
| Left Insula Reactivity | .39 | .93 | .03 | .675 | .963 |  | -.07 | .45 | .00 | .882 | .882 |
| *NS*_change_ x Left Insula Reactivity | 26.70 | 42.49 | .16 | .530 | .764 |  | 9.94 | 19.28 | .05 | .606 | .699 |
| ***Left ACC Reactivity to Negative Emotion*** |  |  |  |  |  |  |  |  |  |  |  |
| *NS*_change_ | -43.39 | 43.13 | -.26 | .314 | .471 |  | -71.05 | 80.96 | -.33 | .380 | .859 |
| Left ACC Reactivity | 1.04 | .96 | .11 | .277 | .554 |  | -.18 | .42 | -.01 | .662 | .882 |
| *NS*_change_ x Left ACC Reactivity | 58.11 | 42.46 | .14 | .171 | .764 |  | -12.79 | 21.74 | -.02 | .556 | .699 |
| ***Left Amygdala Reactivity to Negative Emotion*** |  |  |  |  |  |  |  |  |  |  |  |
| *NS*_change_ | -1.67 | .86 | -.07 | .052 | .156 |  | -.47 | .93 | -.02 | .612 | .859 |
| Left Amygdala Reactivity | -.25 | .16 | -.02 | .112 | .471 |  | -.08 | .22 | -.01 | .730 | .882 |
| *NS*_change_ x Left Amygdala Reactivity | -1.14 | 4.07 | -.02 | .780 | .780 |  | -1.67 | 4.32 | -.02 | .699 | .699 |

*Note.* Youth-reported externalizing and internalizing symptoms were measured by Brief Problem Monitor (BPM). Both adolescent adjustment outcomes were tested in one model, resulting in a total of 6 interaction models (right insula, right ACC, right amygdala, left insula, left ACC, left right amygdala). *p^uc^* represents unadjusted p-values.  *p^c^* represents FDR adjusted *p*-values for the 6 models.
